# The Lark Loop Used for Proximal Biceps Tenodesis: An All-Arthroscopic Technique

**DOI:** 10.1016/j.eats.2022.02.031

**Published:** 2022-06-14

**Authors:** Min Zhou, Chuan-Hai Zhou, Jin-Ming Zhang, Long Yi, Jiang Guo, Jing-Yi Hou, Rui Yang

**Affiliations:** Department of Orthopaedic Surgery, Sun Yat-sen Memorial Hospital, Sun Yat-sen University, Guangzhou, China

## Abstract

Long head of the biceps tendinopathy is a common shoulder problem that is difficult to diagnose and treat. Biceps tenodesis is an effective surgical approach target for long head of the biceps tendon lesions. This article describes an all-arthroscopic proximal biceps tenodesis technique. This technique uses a high-strength suture to construct a tear-resistant loop; fixation is achieved with a suture anchor at the proximal aspect of the intertubercular groove or the greater tuberosity. This tenodesis fixation is simple, with no neurovascular injury or humeral fracture risk. In addition, our technique is cost-effective, with no need for specialty sutures.

Anterior shoulder pain is one of the most common diseases in the field of joint surgery, with an incidence rate of approximately 30%. Long head of the biceps tendinopathy has been recognized as the primary reason for anterior shoulder pain. In about 40% of patients, noticeable pathologic changes are found in the long head of the biceps tendon (LHBT), including tendinitis, tendon wear, SLAP lesions, and partial or complete ruptures of the LHBT.^1^, ^2^, ^3^ In addition, an isolated LHBT lesion is rare, often observed concomitantly with rotator cuff tear or acromial impingement.^4^

It has been reported that tenotomy and tenodesis have equivalent effectiveness regarding relieving the shoulder symptoms caused by LHBT lesions.^5^^,^^6^ However, according to the recent literature, tenodesis might better regain shoulder function and strength and avoid the incidence of cosmetic deformity and cramping when compared with tenotomy.^7^^,^^8^ Hence, the tenodesis technique is more welcomed in the young population and/or patients with a high demand for athletics.^9^ Various surgical methods have been reported for LHBT tenodesis, including both open and arthroscopic fixation with suture anchors, interference screws, and other implants. Most of the different tenodesis patterns showed similar clinical outcomes eventually. Any particular tenodesis pattern can hardly achieve the theoretical native tendon-bone structure. Hence, a secure and straightforward tenodesis approach might be the optimal and appropriate choice for biceps tenodesis.

Duerr et al.^10^ first proposed the “loop ‘n’ tack knot” biceps tenodesis technique, applied in all-arthroscopic LHBT tenodesis, with satisfactory clinical outcomes.^11^ However, this simple technique required specialty sutures, FiberSnare or FiberLink (Arthrex, Naples, FL),^10^^,^^12^ which were commercially unavailable in many regions and limited the further promotion of this technique. Therefore, in this article, we propose a suture technique—the lark-loop technique—that resembles the configuration of the lark’s head knot, based on an alteration of the loop ‘n’ tack technique. The lark-loop technique is characterized by the high capacity of self–anti-sawing tissue, as well as knotless fixation, simplicity, cost-effectiveness, and a broader application market in multiple regions.

## Surgical Technique

### Patient Positioning and Landmark Identification

**Fig 1:**
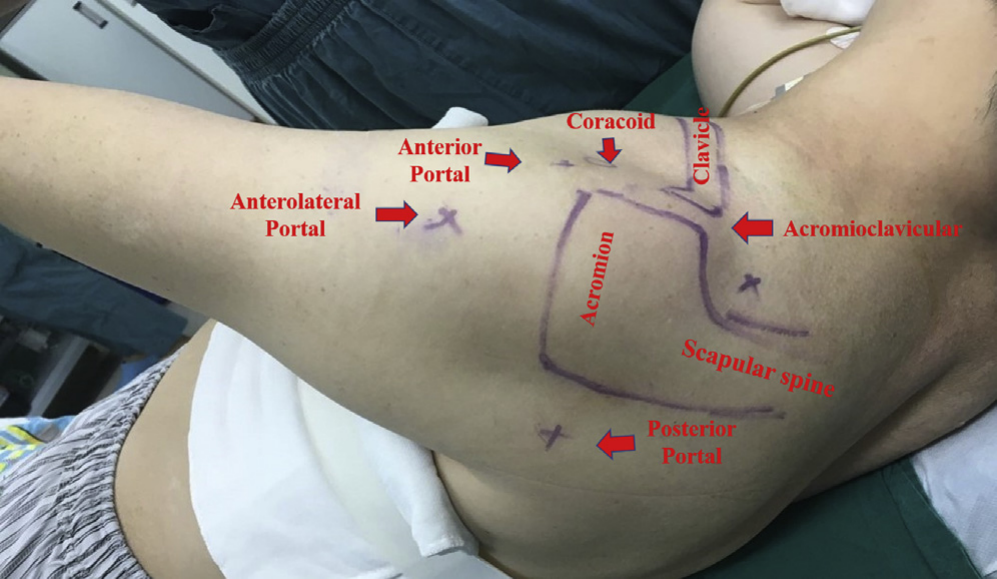
Patient position and landmark identification (left shoulder). The patient is placed in the lateral decubitus position, with continuous affected-extremity traction. The bony landmarks are outlined, and the posterior, anterior, and anterolateral portals are marked.

After receiving general anesthesia combined with a brachial plexus block, the patient is placed in the lateral decubitus or beach-chair position on the operating table, depending on the chief surgeon's preference. In the lateral decubitus position, the operative shoulder is placed in 20° of forward flexion and 35° to 45° of abduction. Continuous traction is applied through the ipsilateral affected upper extremity to gain a larger operative space in the glenohumeral joint. The bony landmarks are identified and marked, together with posterior, anterior, and anterolateral portals (Fig 1).

### Arthroscopic Examination and LHBT Pathology Identification

**Fig 2:**
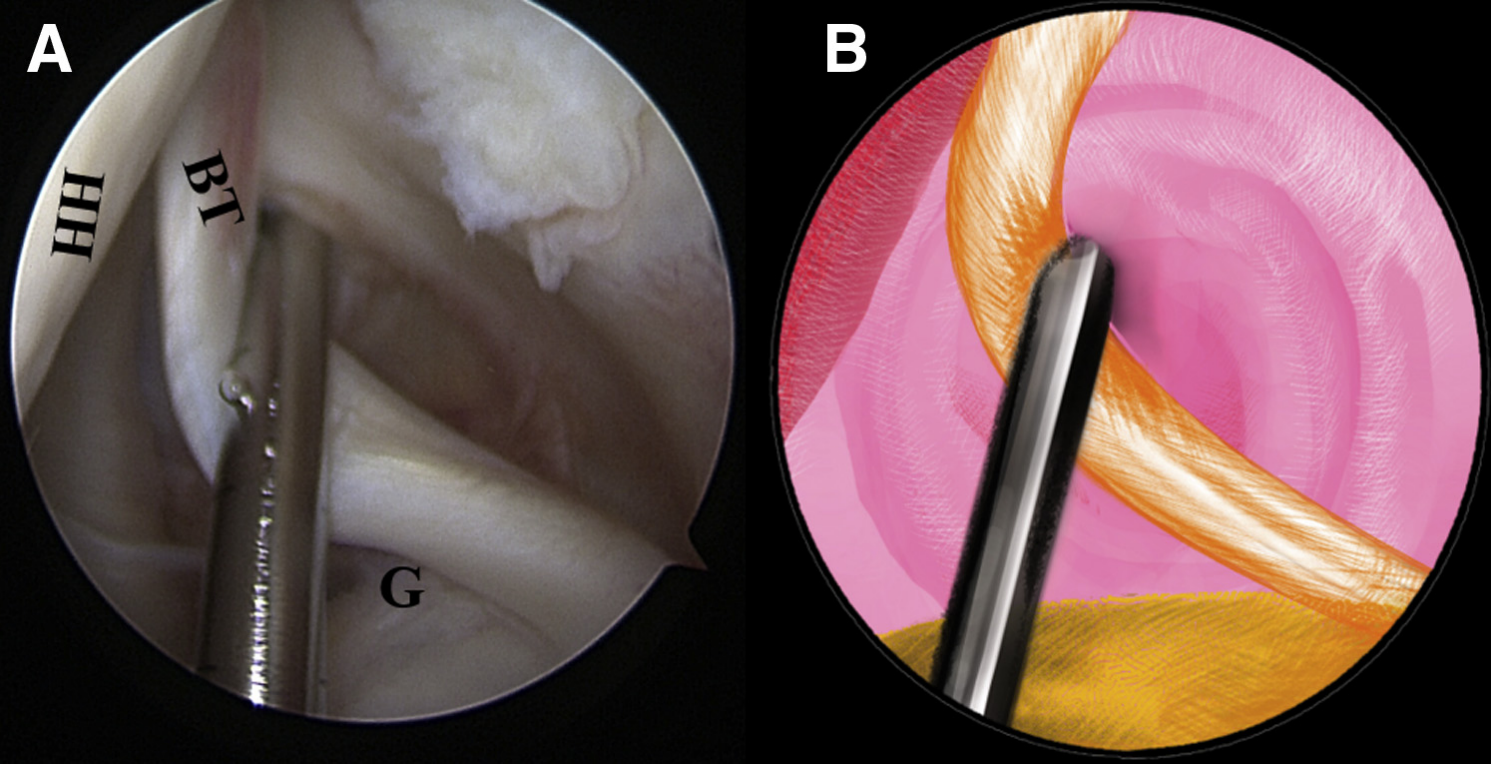
The patient is positioned in the lateral decubitus position. (A) Intra-articular arthroscopic image of a left shoulder from the posterior viewing portal with a 30° arthroscope. A probe from the anterior working portal is used to evaluate the quality of the biceps tendon for an initial assessment. (B) An illustration summarizes the corresponding step. (BT, biceps tendon; G, glenoid; HH, humeral head.).

After a standard posterior portal is established, a 30° arthroscope is inserted into the articular cavity to examine the pathologic lesion of the LHBT thoroughly. To further evaluate the quality of the tendon within the intertubercular groove part, this part of the tendon needs to be pulled toward the intra-articular cavity using a probe from the anterior portal (Fig 2, Video 1).

### Lark-Loop Stitch Construction for LHBT

**Fig 3:**
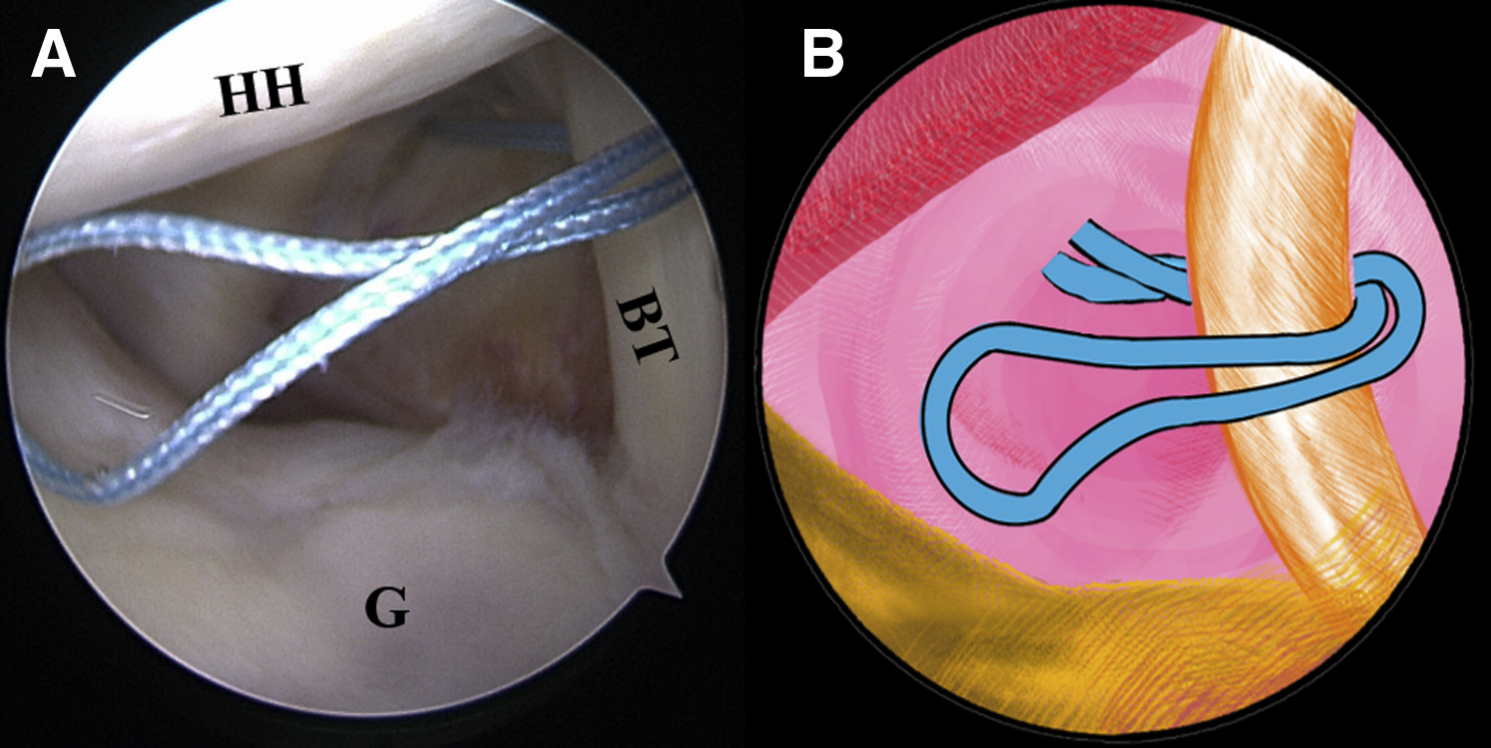
The patient is positioned in the lateral decubitus position. (A) Intra-articular arthroscopic image of a left shoulder from the posterior viewing portal with a 30° arthroscope. A FiberWire is folded in half to be placed at the superior aspect of the biceps tendon through the anterior working portal. (B) An illustration summarizes the corresponding step. (BT, biceps tendon; G, glenoid; HH, humeral head.).

**Fig 4:**
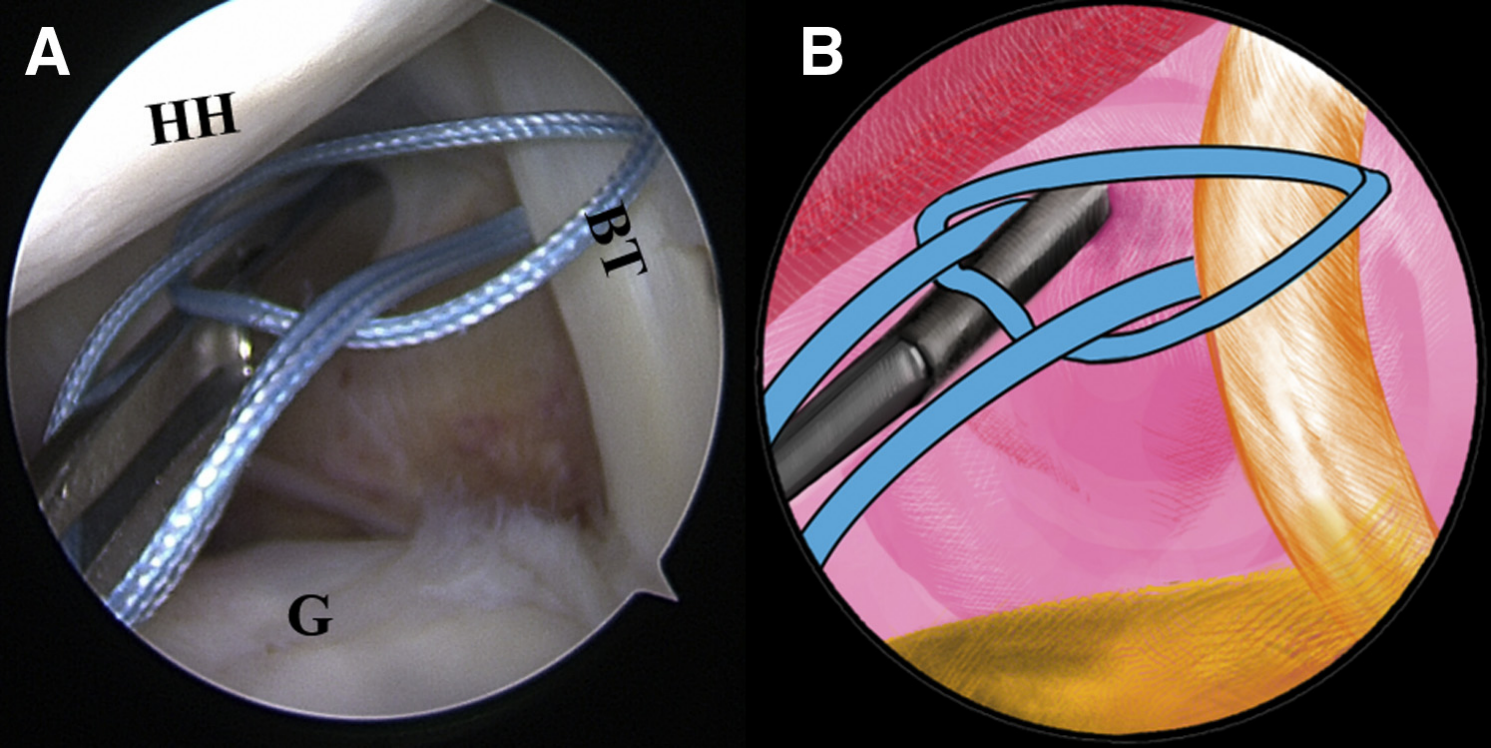
The patient is positioned in the lateral decubitus position. (A) Intra-articular arthroscopic image of a left shoulder from the posterior viewing portal with a 30° arthroscope. Two suture strands are threaded through the loop inferior to the tendon, released, and grasped out of the capsule through the anterior working portal to construct a lark’s head knot. (B) An illustration summarizes the corresponding step. (BT, biceps tendon; G, glenoid; HH, humeral head.).

**Fig 5:**
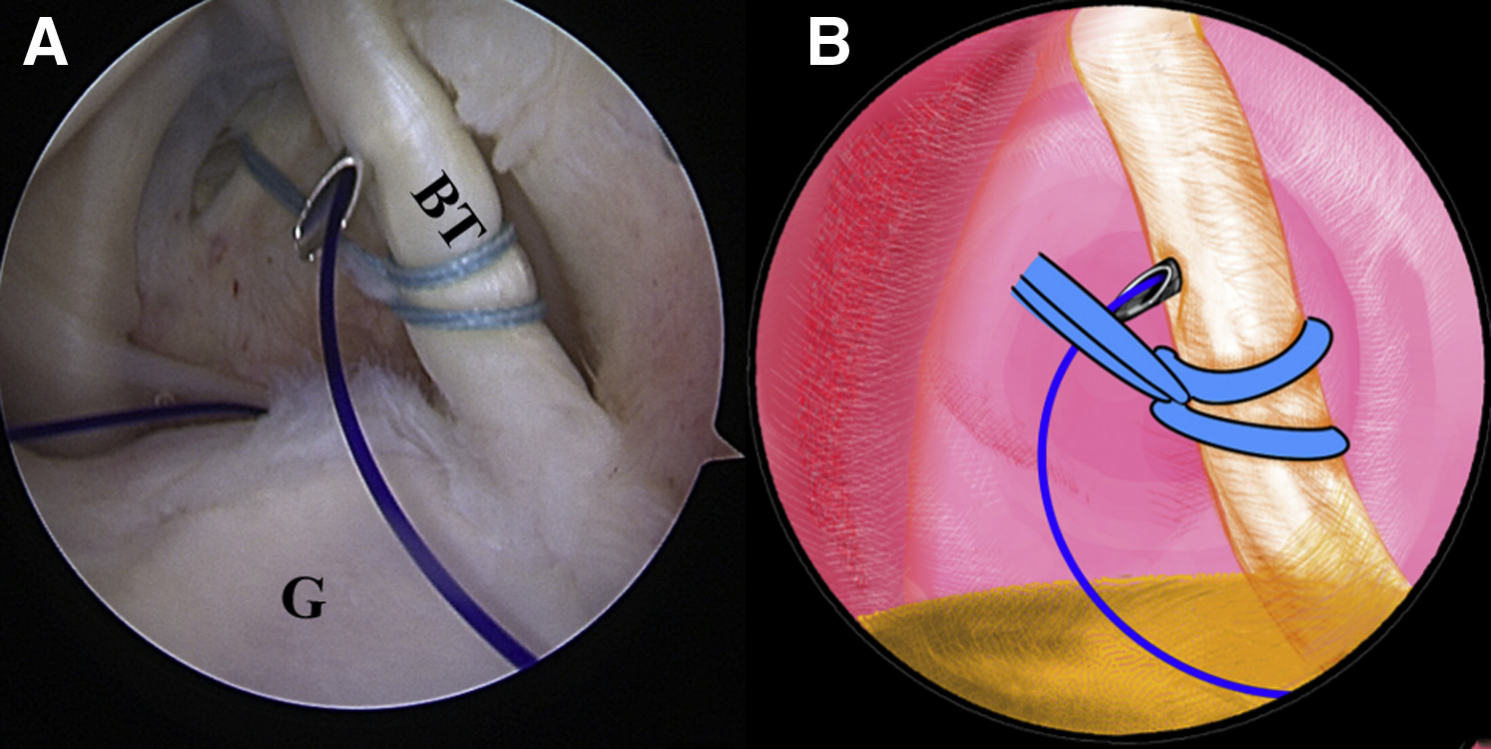
The patient is positioned in the lateral decubitus position. (A) Intra-articular arthroscopic image of a left shoulder from the posterior viewing portal with a 30° arthroscope. An 18-gauge spinal needle is inserted through the middle portion of the tendon, just distal to the knot, to advance a No. 0 PDS II (polydioxanone) suture. (B) An illustration summarizes the corresponding step. (BT, biceps tendon; G, glenoid.).

**Fig 6:**
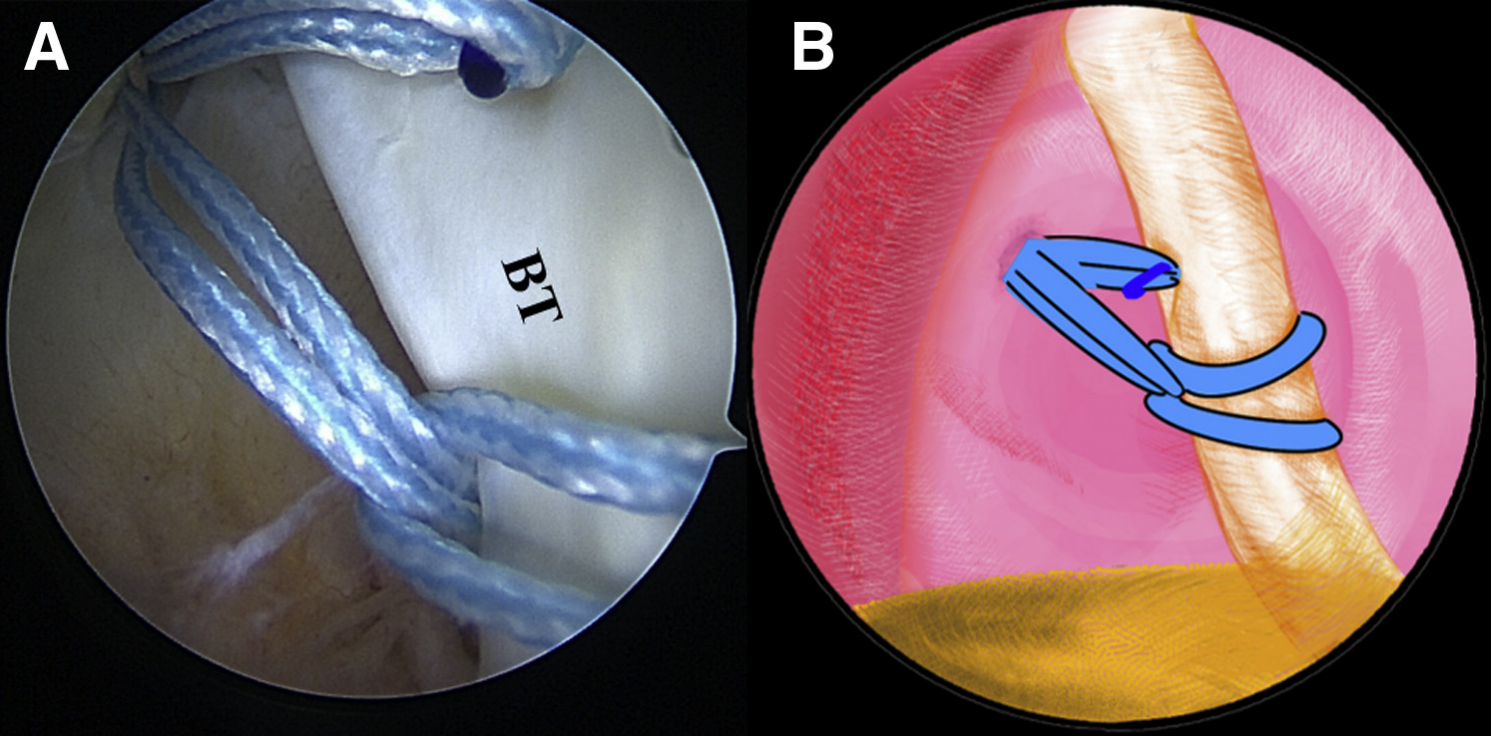
The patient is positioned in the lateral decubitus position. (A) Intra-articular arthroscopic image of a left shoulder from the posterior viewing portal with a 30° arthroscope. An overhand knot has been tied on the 2 strands of the FiberWire ends with the polydioxanone suture. The spinal needle is retrieved, and the polydioxanone suture inside is pulled out, helping to shuttle the 2 strands of FiberWire ends through the tendon. (B) An illustration summarizes the corresponding step. (BT, biceps tendon.).

**Fig 7:**
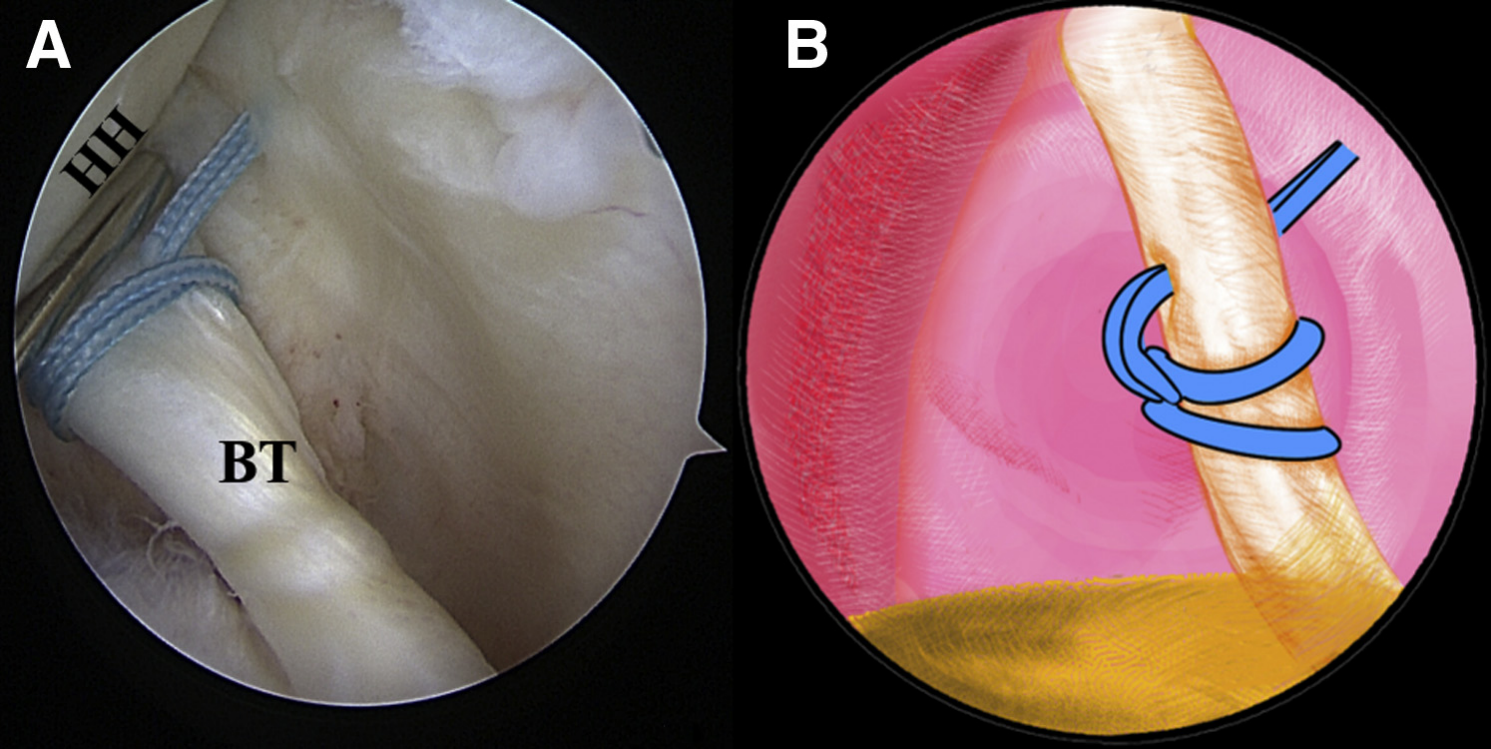
The patient is positioned in the lateral decubitus position. (A) Intra-articular arthroscopic image of a left shoulder from the posterior viewing portal with a 30° arthroscope. Final completion of the lark loop is shown. (B) An illustration summarizes the lark-loop placement on the biceps tendon. (BT, biceps tendon; HH, humeral head.).

Once the LHBT tenodesis is confirmed, a No. 2 FiberWire suture (Arthrex) is folded in half and inserted into the capsule with a suture grasper from the anterior portal. The suture loop is first placed at the superior aspect of the LHBT (Fig 3, Video 1). Afterward, 2 suture strands are threaded through the loop inferior to the tendon, released, and grasped out of the capsule from the anterior portal to construct a lark’s head knot on the LHBT (Fig 4, Video 1). The location of the lark’s head knot can be optionally adjusted based on the ultimate tension of the biceps tendon. When the site of the lark’s head knot is confirmed, an 18-gauge spinal needle is inserted through the middle portion of the tendon, just distal to the knot, to advance a No. 0 PDS II (polydioxanone) suture (Ethicon [Johnson & Johnson], Somerville, NJ) as a guiding suture (Fig 5, Video 1). Subsequently, the end of the PDS suture is grasped out of the capsule with the grasper through the anterior portal to tie an overhand knot on the 2 suture strands, while the other end of the PDS suture within the spinal needle is held still by an assistant. Finally, the spinal needle is retrieved, and the PDS suture inside is pulled out, helping to shuttle the 2 strands of FiberWire through the tendon (Fig 6, Video 1). A self-locking and highly resistant loop configuration is constructed (Fig 7, Video 1).

### Long Head of Biceps Tenodesis

**Fig 8:**
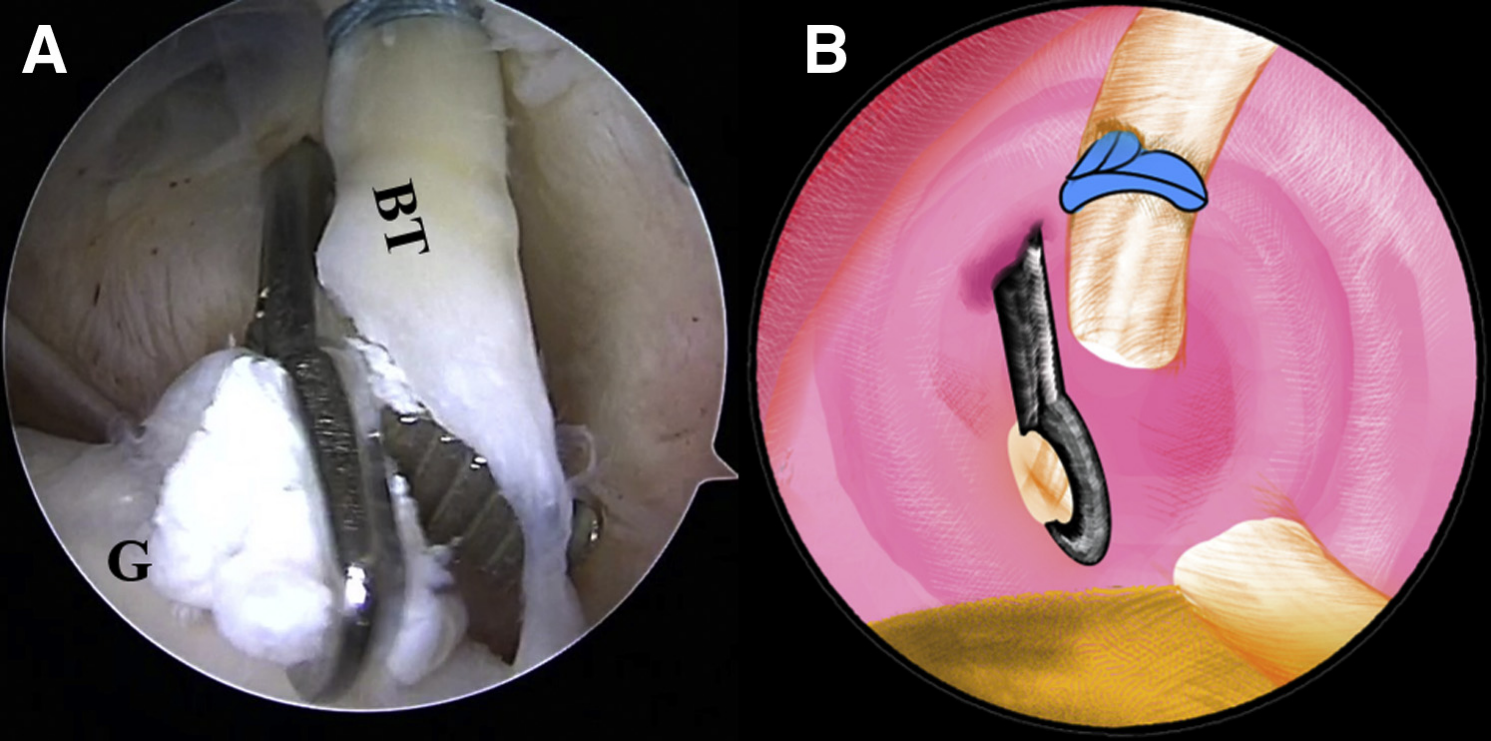
The patient is positioned in the lateral decubitus position. (A) Intra-articular arthroscopic image of a left shoulder from the posterior viewing portal with a 30° arthroscope. The LHBT is detached from its insertion site on the superior-labral junction with a punch forceps through the anterior working portal. (B) An illustration summarizes the corresponding step. (BT, biceps tendon; G, glenoid.).

**Fig 9:**
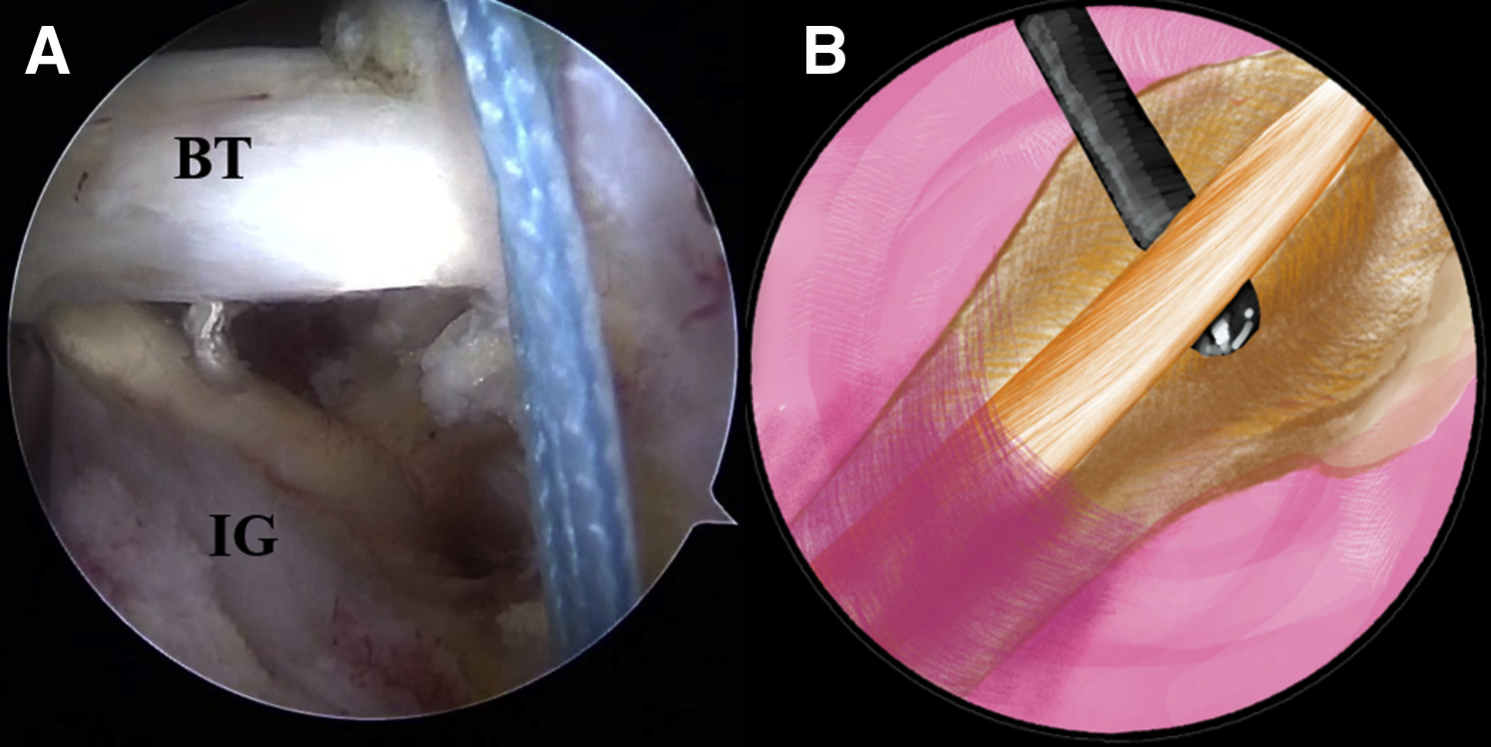
The patient is positioned in the lateral decubitus position. (A) Arthroscopic image in the subacromial space of a left shoulder from the anterolateral viewing portal with a 30° arthroscope. The stump of the biceps tendon is pulled out of the articular cavity. (B) An illustration summarizes the corresponding step. (BT, biceps tendon; IG, intertubercular groove.).

**Fig 10:**
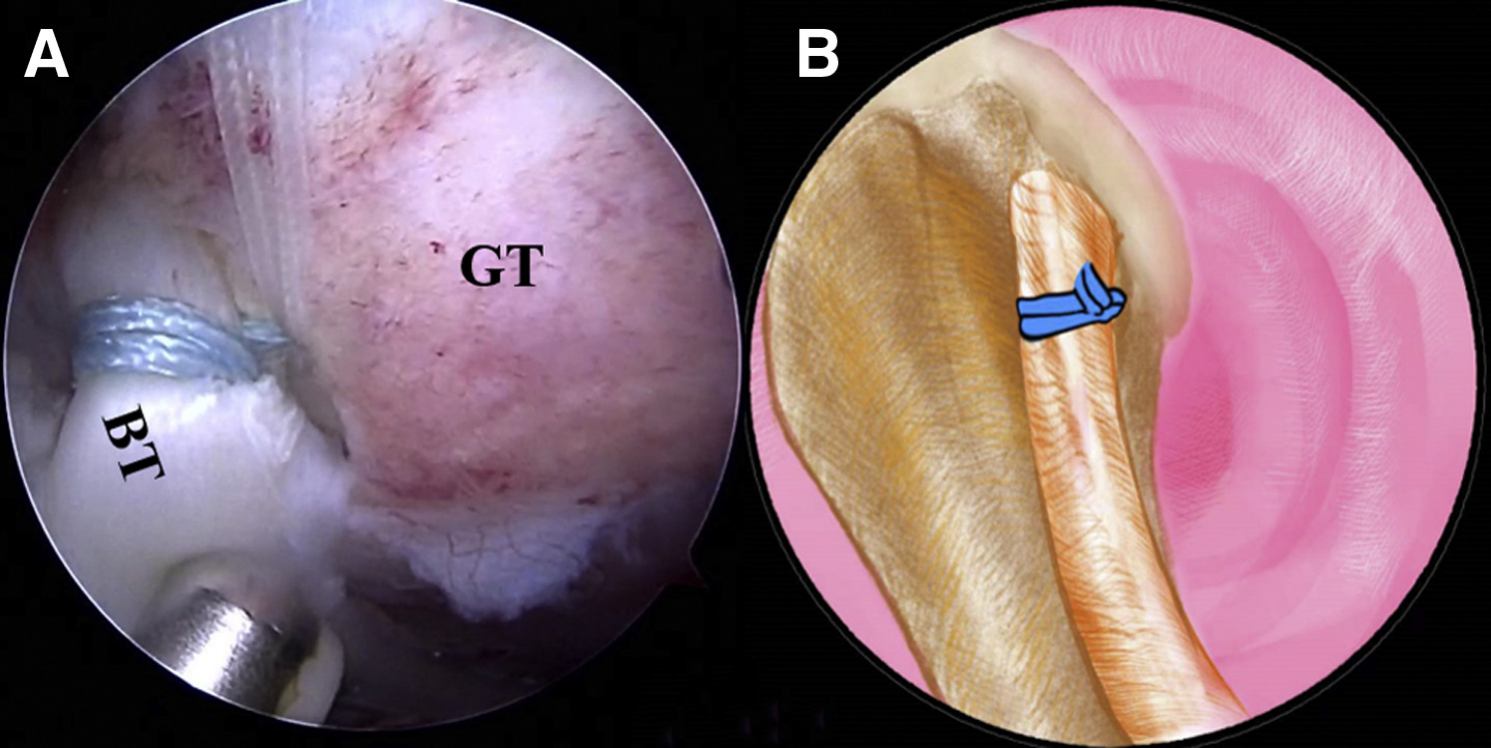
The patient is positioned in the lateral decubitus position. (A) Arthroscopic image in the subacromial space of a left shoulder from the anterolateral viewing portal with a 30° arthroscope. The strands of the lark loop are co-anchored with the lateral row during rotator cuff repair with a 4.75-mm SwiveLock C anchor. (B) An illustration summarizes the strands of the lark loop firmly anchored at the intertubercular groove in the isolated biceps tenodesis with a 4.75-mm SwiveLock C anchor. (BT, biceps tendon; GT, greater tuberosity.).

The LHBT is detached from its insertion site on the superior-labral junction with a punch forceps (Fig 8, Video 1). Under these circumstances, the surgeon should make sure there is enough distance between the lark loop and the stump of the tendon in case the loop slips off. The arthroscope is subsequently moved to the subacromial space. The transverse ligament is completely released and the LHBT is thoroughly exposed in the intertubercular groove with radiofrequency ablation through the anterolateral portal. The stump of the biceps tendon is pulled out of the articular cavity (Fig 9, Video 1). Thorough debridement of the tendon sheath is performed to visualize the bony portion of the intertubercular groove. The intended anchor site is refreshed with an arthroscopic burr to facilitate later tendon-bone healing. A pilot hole is drilled with a punch for the 4.75-mm SwiveLock C anchor (Arthrex) at the intertubercular groove close to the capsule border perpendicularly. Two strands of the lark loop are loaded into the eyelet of the anchor. Finally, the anchor is placed into the pilot hole, with all slack sutures completely pulled out (Fig 10B). In addition, the suture can be co-anchored with the anchor of the lateral row in the rotator cuff repair when concomitant with a rotator cuff tear (Fig 10A, Video 1).

### Postoperative Rehabilitation

The patient undergoes shoulder and elbow brace immobilization immediately after the operation. Passive activity of the elbow and wrist should be started on the second day and continue until 6 weeks after the operation. To promote tendon-to-bone healing, active elbow flexion is prohibited within 6 weeks after the operation. Active elbow flexion rehabilitation starts after 6 weeks and is gradually increased until 3 months postoperatively. Biceps strength training is restored after 3 months postoperatively.

## Discussion

The optimal treatment for the patients with symptomatic LHBT lesions remains controversial.^13^ At present, tenotomy and tenodesis of the LHBT are still the recommended choices.^5^ However, tenodesis has displayed prominent superiority in maintaining the length-tension relation of the biceps, which helps to reduce the incidence of Popeye deformity.^7^ Thus, tenodesis seems to have gained more popularity than tenotomy. The pattern and location of biceps tenodesis largely depend on the surgeon’s experience and preference when it comes to the specific tenodesis technique.

Multiple studies have proved that arthroscopic tenodesis and open tenodesis have similar clinical outcomes. A kind of all-arthroscopic tenodesis—the loop ‘n’ tack technique—was first reported by Duerr et al.^10^ using a FiberSnare. Later, Acosta et al.^12^ conducted an in vitro biomechanical study and found that the loop ‘n’ tack method had an ultimate load to failure similar to that of the Krackow stitch. Therefore, the loop ‘n’ tack technique has proved safe and effective. However, a FiberSnare or FiberLink is a necessary surgical supply for the loop ‘n’ tack suture and is unavailable in many areas. For this reason, we made some alterations. This article describes a modified loop ‘n’ tack biceps tenodesis technique—the lark-loop technique—a simple, knotless, tear-resistant, and all-arthroscopic tendon fixation technique without any specialty sutures. First, a lark knot is tied on the LHBT with FiberWire in place of the looped hitch in the loop ‘n’ tack technique. Second, a spinal needle within a PDS suture, rather than an arthroscopic tissue penetrator, is pierced through the tendon distal to the lark knot to help shuttle the 2 suture limbs through the LHBT. We believe that the spinal needle has unique superiority in avoiding aggravation of the LHBT injury during tendon penetration. Technique-related tips and tricks are summarized in Table 1.

**Table 1:** Tips and Tricks

A proper anterior portal will facilitate access above and below the LHBT.
The anterior portal should not be close to the exit of the LHBT in the articular cavity.
A spinal needle is used to penetrate through the biceps tendon, rather than a penetrator, to avoid aggravating LHBT injury.
The loop should be placed as close as possible to the labrum for hypotonic tendon fixation.
Thorough debridement of the intertubercular groove should be conducted to prevent residual pain.
The suture of the loop should be >1 cm from the edge of the incision to avoid pulling out the suture.

LHBT, long head of biceps tendon.

The most attractive advantage of the all-arthroscopic tenodesis technique presented in this article is cost-effectiveness: Except for conventional high-strength sutures and suture anchors, no additional specialty sutures or implants are needed for tenodesis. Moreover, just like the loop ‘n’ tack technique, the lark-loop technique is a time-saving method that can be accomplished within several minutes. Furthermore, the safety of this surgical technique is guaranteed. The entire surgical operation is accomplished by direct vision with the arthroscopic. Similarly to other suprapectoral or in-the-groove tenodesis techniques, there is no risk of neurovascular injury or proximal humeral fracture. In addition, the lark-loop tenodesis technique is accomplished using a knotless pattern that does not require any knot-tying procedures. The knotless suture anchor eliminates knot-related side effects, such as knot irritation, knot loosening, and knot migration. Moreover, this biceps tenodesis technique offers ease of replication and excellent visualization. As a result, the lark-loop technique can be mastered quickly by surgeons.

One of the potential disadvantages of the described technique is the possible risk of suture anchor pullout, leading to tenodesis failure. Besides, in this technique, the biceps tendon docks at the surface of the humeral cortex with the use of a knotless suture anchor. Therefore, the effective contact area of tendon to bone is questionable. There are limited studies evaluating the contact pressure and contact area between knotless and knotted biceps tenodesis techniques. We agree with the concern of Su et al.^14^ that the lack of effectual contact will delay the process of tendon-to-bone healing. Advantages and disadvantages of our technique are listed in Table 2.

**Table 2:** Advantages and Disadvantages

Advantages
Our operative technique using all-arthroscopic visualization can reduce the probability of intraoperative neurovascular injury and postoperative infection.
This technique reduces unnecessary extra incisions and the need for specialty sutures or instruments.
The knotless technique reduces potential adverse effects regarding knotted suture anchors.
The anchor site is flexible.
This technique is simple to perform and easy for surgeons to learn and repeat.
This technique is a time-saving tenodesis technique.
Disadvantages
New practice cure is necessary for operators.
If the suture of the loop is too close to the edge of the biceps incision, the proximal biceps can be removed during or after surgery.
There is a possible risk of suture anchor pulling out.
The onlay knotless suture anchor tenodesis technique might result in insufficient contact surface for the tendon-to-bone healing.
Clinical follow-up data are lacking.

In general, the lark-loop biceps tenodesis technique is a safe, cost-effective technique that can be performed as an alternative to the loop ‘n’ tack technique. However, further biomechanical studies and clinical trials are needed to evaluate the biomechanical properties of this tenodesis technique and the clinical outcome of the tendon-bone fixation.

## Supplementary Data

ICMJE author disclosure forms

Video 1 Lark-loop technique for biceps tenodesis. The patient is in the lateral decubitus position, and visualization of the left shoulder starts in the posterior portal with a 30° arthroscope. A lark’s head knot is tied on the biceps tendon with a No. 2 FiberWire suture. The 2 suture strands are shuttled through the biceps tendon distal to the lark’s head knot. The biceps tendon is detached from its insertion site. Then, the arthroscope is moved into the subacromial space. Thorough debridement is performed at the intertubercular groove, and the biceps tendon is exposed. Next, the stump of the tendon is pulled out of the articular cavity. Finally, the tendon can be fixed at the greater tuberosity concomitant with the rotator cuff repair or the intertubercular groove in isolated tenodesis of the long head of the biceps tendon with a 4.75-mm SwiveLock C anchor. The critical steps of the technique are summarized in the illustrations.
